# Tumor-associated oncogenes go on (phage) display

**DOI:** 10.18632/oncotarget.112

**Published:** 2010-06-12

**Authors:** Eugene Kandel

**Affiliations:** Department of Cell Stress Biology, Roswell Park Cancer Institute, Buffalo, NY

The immune system has a long-recognized ability to target select proteins produced by tumor cells. Such tumor-specific antigens may be uniquely present in cancer as a result of mutations or abnormal protein modifications. In other cases, the increased immunogenicity of structurally normal proteins is less obvious and may represent, albeit not always, elevated levels of their expression in malignant cells. Immune response to tumor-specific antigens raises two important questions: could it be selectively enhanced for a therapeutic effect, and could it be used to discover the actual antigens in order to understand the properties of the tumors and to diagnose and classify the disease? The thought-provoking report by Ionov in the current issue of this journal offers news insights into advancing both of these issues [1].

The experimental approach (See Figure) relies on the use of a phage-display library, which is first depleted of the phages that bind to control antibodies, such as the ones obtained from a healthy donor, and then is subjected to precipitation using an antibody fraction from a cancer patient. The phages enriched at this step are expected to display the peptides that are recognized by patient's, but not control's antibodies. The unbound phages are eliminated, while the precipitated ones could be rescued in bacteria. This sequence of steps could be repeated multiple times for further enrichment. Subsequently, the sequence of the presented peptides is determined by sequencing the enriched phages. These data are examined for homology to known human proteins. Not surprisingly, the short peptides reveal homology to numerous proteins. The picture is further complicated by an obvious fact that some epitopes may not be continuous, or may include posttranslational modification and, hence, would not be detected in a trivial homology search. The key assumption that Ionov made is that an immunogenic protein is likely to be targeted by multiple antibodies, and at least some of them would have continuous epitopes. If this is the case, one may expect the same protein identified in the searches for homology with multiple selected peptides. Indeed, the report gives several examples of such proteins. At least one of them, fucosyltransferase 6, was confirmed to be overexpressed in the tumor from the patient who was the donor of the respective serum sample.

**Fig.**
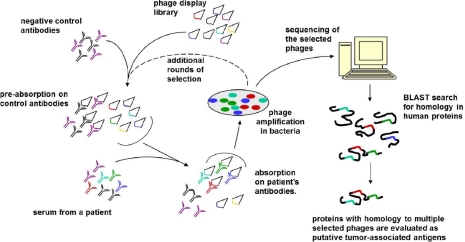
The Experimental Approach

The idea to use phage libraries as means of interrogating cancer-directed immunity is not entirely new. However, the prior work relied on a cumbersome strategy of choosing a single phage-displayed peptide and using it to raise rabbit polyclonal antibodies [2]. The assumption was that the peptide would be a good enough mimic of the original protein, so that the antibodies raised against the former could be used to identify the latter. Although successfully used in the original study, this strategy is not readily scaled up for the analysis of multiple peptides. Also, the choice of an individual peptide to pursue is not an easy one: if it is made in favor of a peptide, which is consistently bound by immunoglobulins from multiple patients, less commonly targeted epitopes have to be ignored, even if they all actually correspond to one protein.

A simple and elegant solution proposed by Ionov is to rely on bioinformatics to look for candidate proteins, even at the risk of ignoring the peptides which do not mimic linear epitopes. Despite some obvious inherent limitations, this promises to be a very fruitful approach, as it is readily interfaced with high-throughput sequencing and could be used to pick proteins that are targeted through distinct epitopes either in a single patient or in different individuals. The next natural step is to expand this methodology to a larger patient population and to various cancer types in an attempt to identify the signature of common antigens, which could be used for diagnostic purposes and might yield some clues to the molecular pathology of the disease. Another important issue is whether the reactivity to the identified antigens changes predictably during growth, remission and recurrence of the disease. If this is indeed the case, one might be able to select a set of peptides that are recognized by tumor-specific antibodies in a given patient and could be used to probe the state of the disease using a simple blood test. Potential complication reside in the choice of negative control antibodies, which are used for pre-absorption of the phage library, and discriminating between tumor-specific antigens and any additional auto-antigens that may arise in an individual. It is also tempting to compare the sequences of the selected peptides to the databases of viral proteins, as an attempt to examine the possible viral contribution to the disease.

While the tumor-associated antigens revealed by immuno-profiling may be highly significant for diagnostic purposes, one has to be cautious not to exaggerate their mechanistic roles in disease progression. Such antigens may be mere byproducts of engaged oncogenic pathways or purely serendipitous variations in cancer cells. In this aspect, immuno-profiling strategy of Ionov joins other methods of comparative analysis, which complement, rather than replace the function-based gene discovery techniques (discussed elsewhere [3-4]) and other conventional techniques of molecular oncology.

Certainly, a large number of tumor-associated antigens are not recognized as continuous epitopes. At this time, one cannot conclude whether the omission of such epitopes from consideration does or does not critically impair the technology. In fact, for as long as the interaction of these epitopes with antibodies is faithfully mimicked by the selected peptides, those peptides might be useful in conjunction with appropriate adjuvants and chemotherapy to boost the anti-tumor activity of the patient's immune system (as is done with conventional peptide cancer vaccines [5]) even if the actual antigen remains unknown. The utility of this approach is yet to be tested and has to be evaluated against the competing technologies, such as whole-cell tumor vaccines [6].
